# Myostatin Promotes Interleukin-1β Expression in Rheumatoid Arthritis Synovial Fibroblasts through Inhibition of miR-21-5p

**DOI:** 10.3389/fimmu.2017.01747

**Published:** 2017-12-08

**Authors:** Sung-Lin Hu, An-Chen Chang, Chien-Chung Huang, Chun-Hao Tsai, Cheng-Chieh Lin, Chih-Hsin Tang

**Affiliations:** ^1^Program for Aging, China Medical University, Taichung, Taiwan; ^2^Department of Family Medicine, China Medical University Hospital, Taichung, Taiwan; ^3^Institute of Biomedical Sciences, National Chung Hsing University, Taichung, Taiwan; ^4^Division of Immunology and Rheumatology, Department of Internal Medicine, China Medical University Hospital, Taichung, Taiwan; ^5^Graduate Institute of Clinical Medical Science, China Medical University, Taichung, Taiwan; ^6^School of Medicine, China Medical University, Taichung, Taiwan; ^7^Department of Orthopedic Surgery, China Medical University Hospital, Taichung, Taiwan; ^8^Department of Medical Research, China Medical University Hospital, Taichung, Taiwan; ^9^Graduate Institute of Basic Medical Science, China Medical University, Taichung, Taiwan; ^10^Department of Biotechnology, College of Health Science, Asia University, Taichung, Taiwan

**Keywords:** myostatin, interleukin-1β, miR-21-5p, mitogen-activated protein kinase, rheumatoid arthritis

## Abstract

Rheumatoid arthritis (RA) is characterized by the infiltration of a number of pro-inflammatory cytokines into synovial fluid and patients with RA often develop joint destruction and deficits in muscle mass. The growth factor myostatin is a key regulator linking muscle mass and bone structure. We sought to determine whether myostatin regulates rheumatoid synovial fibroblast activity and inflammation in RA. We found that levels of myostatin and interleukin (IL)-1β (a key pro-inflammatory cytokine in RA) in synovial fluid from RA patients were overexpressed and positively correlated. In *in vitro* investigations, we found that myostatin dose-dependently regulated IL-1β expression through the ERK, JNK, and AP-1 signal-transduction pathways. Computational analysis confirmed that miR-21-5p directly targets the expression of the 3′ untranslated region (3′ UTR) of IL-1β. Treatment of cells with myostatin inhibited miR-21-5p expression and miR-21-5p mimic prevented myostatin-induced enhancement of IL-1β expression, showing an inverse correlation between miR-21-5p and IL-1β expression during myostatin treatment. We also found significantly increased paw swelling in an animal model of collagen-induced arthritis (CIA), compared with controls; immunohistochemistry staining revealed substantially higher levels of myostatin and IL-1β expression in CIA tissue. Our evidence indicates that myostatin regulates IL-1β production. Thus, targeting myostatin may represent a potential therapeutic target for RA.

## Introduction

The key features of rheumatoid arthritis (RA), a chronic autoimmune disease, include synovial inflammation and articular cartilage destruction (1). RA synovial fibroblasts (RASFs) in the superficial layer of the synovium are implicated in the initiation and progression of RA disease (2). Activated RASFs upregulate tumor necrosis factor-alpha (TNF-α) and interleukin-1 beta (IL-1β) expression, resulting in bone resorption during RA disease (3). Therefore, it is important to determine how to influence synovial fibroblast inflammation.

Interleukin-1β is a key pro-inflammatory cytokine in autoimmune diseases, such as RA (4); it is implicated in type 1 diabetes mellitus (5) and appears to contribute to disease severity in experimental autoimmune encephalomyelitis (6). It is known that IL-1β production is highly increased in RA plasma and synovium, correlating with disease activity (6). Biological therapeutics have been designed to target IL-1β or its receptor in the treatment of RA. For instance, the human anti-IL-1β monoclonal antibody canakinumab neutralizes IL-1β signaling and thereby suppresses inflammation in patients (7). Moreover, anakinra (a recombinant human IL-1β receptor antagonist), alone or combination with methotrexate, significantly reduces the severity of RA as compared with placebo (8, 9). This evidence demonstrates that IL-1β serves as a key mediator in RA pathogenesis and that blocking IL-1β or its receptor delays RA disease progression.

Overexpression of pro-inflammatory cytokines in synovial fluid is a feature of RA disease. Accumulating evidence shows that miRNAs are influential upstream mediators of pro-inflammatory cytokines, such as miR-138-5p, which regulates IL-1β-induced extracellular matrix degradation of cartilage (10–12). It has also been reported that miRNAs contribute to RA pathogenesis by upregulating monocyte chemokine production and the expression of pro-inflammatory chemokine receptors, promoting inflammatory cell recruitment and retention in RA synovium (13). Importantly, the downregulation of miR-21 in RA disease correlates with the expression and activation of STAT-3, a process that promotes Th17 cell differentiation and results in an imbalance of Th17 and Treg cells (14, 15). Hence, it is useful to determine whether miRNAs are involved in the RA inflammation pathway.

The myokine myostatin (also termed growth differentiation factor 8, or GDF-8), secreted growth and differentiation factor, belongs to the transforming growth factor-β superfamily (16). Myostatin is produced and secreted from myocytes and inhibits myogenesis, inducing muscle atrophy (17). Levels of myostatin are positively correlated with pro-inflammatory factors in both chronic kidney disease and type 2 diabetes (18, 19), and a high expression of myostatin has been found in RA synovial membranes. Myostatin deficiency and treatment with a myostatin monoclonal antibody diminishes inflammation, osteoclast differentiation, and bone erosion in a murine RA disease model (20). It appears that myostatin in implicitly involved in the progression of RA disease. However, it is unclear as to whether myostatin directly leads to RASF inflammation. Although myostatin is widely understood to be a key regulator of pro-inflammatory cytokines in many diseases, its role during the progression of RA remains uncertain. We demonstrate that both myostatin and IL-1β are overexpressed in synovial fluids from RA patients and in tissue from collagen-induced arthritis (CIA) mice. This present study demonstrates that myostatin directly induces IL-1β production *via* the ALK receptor, JNK, ERK, and AP-1 signaling pathways, as well as *via* downregulation of miR-21-5p expression. We suggest that our findings help to elucidate the mechanisms of the myostatin/IL-1β axis and will improve our understanding of how to better treat RA disease.

## Materials and Methods

### Materials

We purchased IL-1β, JNK, p-JNK, ERK, p-ERK, c-Jun, and p-c-Jun primary antibodies from Santa Cruz Biotechnology, myostatin-specific anti-rabbit polyclonal antibody from Abcam and recombinant human myostatin from PeproTech. R&D Systems supplied the myostatin and IL-1β enzyme-linked immunosorbent assay (ELISA) kits. Calbiochem supplied the inhibitors for ALK4/5/7 (SB431542), ERK (U0126), JNK (SP600125), and AP-1 (Curcumin and Tanshinone IIA). All other chemicals were purchased from Sigma-Aldrich.

### Cell Culture

We cultured the human rheumatoid cell line (MH7A; Riken cell bank, Ibaraki, Japan) in RPMI-1640 medium that was supplemented with 10% fetal bovine serum (FBS) and penicillin/streptomycin 100 U/mL. Cell incubation was performed in a humidified atmosphere of 37°C, 5% CO_2_. We changed the culture medium on alternate days and passaged the cells when they had grown to 80% confluence.

The inhibitors for ALK4/5/7 (SB431542), ERK (U0126), JNK (SP600125), and AP-1 (curcumin and tanshinone IIA) were used to study the signaling transduction pathway. We cultured cells in 6-well plates with RPMI-1640 media containing 10% FBS. Cells were grown to 70% confluence, then incubated overnight in serum-free medium and treated for 30 min with or without the inhibitors for ALK4/5/7, ERK, JNK, and AP-1 (all at the concentration of 10 µM), followed by myostatin (10 ng/mL). Cells were harvested and examined by western blot, ELISA, and immunofluorescence assays.

### Preparation of Nuclear Extracts

MH7A cells were incubated with myostatin, harvested with centrifugation then resuspended for 10 min in hypotonic buffer A (21) on ice. Nuclei were isolated by centrifugation at 12,000*g* for 5 min. The nuclear pellet was suspended in buffer C (21) on ice for 30 min then vortexed for 15 s. The mixture was centrifuged at 12,000*g* for 10 min to collect supernatants containing nuclei proteins, then stored at −70°C.

### Human Synovial Fluids

Study approval was granted by China Medical University’s ethics committee and all subjects submitted written informed consent prior to entering the study. The study protocol was approved by China Medical University Hospital’s Institutional Review Board. Samples of synovial fluids were taken during total knee arthroplasty in 15 patients aged 40–60 years [10 patients with RA and 5 with osteoarthritis (OA)], then stored at −80°C until assay by myostatin and IL-1β ELISA kits.

### mRNA and miRNA Quantification

We used a TRIzol kit (MDBio) to extract total RNA from MH7A cells and the Mir-XTM miRNA First Strand Synthesis Kit to quantify miRNAs; we followed the manufacturers’ protocols for both procedures. Each sample was evaluated for RNA quality and yield, using the A260/A280 ratio of 2:1, with the NanoVue spectrophotometer (GE Healthcare). Using the M-MLV Reverse Transcriptase kit (Invitrogen), we synthesized complementary DNA from 1 µg of total RNA and the KAPA SYBR^®^ FAST qPCR Kit (Applied Biosystems) for real-time quantitative polymerase chain reaction (qPCR) analysis under the following cycling conditions: 10 min at 95°C, then 40 cycles at 95°C for 15 s, and 60°C for 60 s. The relative expression of IL-1β and miRNA was normalized to endogenous GAPDH and snRNA U6, respectively, as internal controls. We used the threshold cycle (*CT*) to calculate the relative expression levels (22).

### ELISA Assay

Downstream signaling pathways involved in myostatin stimulation were investigated. Human MH7A cells were cultured and pretreated with the pathway inhibitors (as indicated earlier), followed by myostatin treatment. We collected the supernatant medium as conditioned medium (CM) and stored it at −80°C until assay. The IL-1β ELISA kit assayed IL-1β in the CM, as per protocol.

### Western Blotting Protocol

Proteins were electrophoresed in sodium dodecyl sulfate-polyacrylamide resolving gel and transferred to Immobilon polyvinyldifluoride membranes, which were incubated in 4% bovine serum albumin (BSA) as blocking buffer for 1 h at room temperature, then probed overnight with primary antibodies (1:3,000) at 4°C. After three washes, the membranes were incubated (for 1 h at room temperature) with anti-rabbit peroxidase-conjugated secondary antibody (1:3,000). The membranes were developed *via* enhanced chemiluminescence then detected using a LAS-4000 image reader (FujiFILM).

### Plasmid Construction and Luciferase Assays

The DNA fragment was constructed by RT-PCR with the following thermal cycling conditions: 3 min at 94°C once, then 35 cycles at 94°C for 1 min, 54°C for 1 min, and 72°C for 2 min. The primers 5′-AACCGCTTCCCTATTTATTT-3′ and 5′-GCTCATTTATAAATATTCCC-3′ were used to construct a DNA fragment of human IL-1β 3′-UTR containing the miR-21-5p binding site. This was inserted into a pGLO-2 basic vector and the construct was confirmed by DNA sequencing (Applied Biosystems 3730xl DNA analyzer). The plasmid containing mutant-IL-1β 3′-UTR was purchased fromGENEWIZ.

The MH7A cells were transfected with plasmid (1 µg), using Lipofectamine 2000 (Invitrogen), as per protocol. After applying myostatin for 24 h, we added reporter lysis buffer (Promega) to each well and scraped the cells, which were centrifuged at 13,000 rpm for 2 min to collect the supernatant. Aliquots of cell lysates were plated into a 96-well opaque, black plate; luciferase substrate was added to all aliquots and luminescence was recorded using a microplate luminometer.

### Immunofluorescence

We cultured MH7A cells on 12-mm coverslips in 24-well plates in RPMI-1640 supplemented with 10% FBS. At 70% confluence, cells were incubated as previously indicated then fixed with 3.7% paraformaldehyde at room temperature for 20 min and washed twice with PBS. Triton X-100 (0.1%) in PBS was added to the mixture for 5 min at room temperature, then the cells were washed three times with PBS and incubated with anti-c-Jun (1:100) at 4°C and left overnight. After three PBS washes, the cells were incubated for 1 h with fluorescein isothiocyanate conjugated secondary antibody. Nuclei were stained with DAPI and cells were mounted and detected by fluorescence microscopy (Zeiss).

### Chromatin Immunoprecipitation (ChIP) Assay

Cells were cultured in RPMI-1640 with 10% FBS. At 70% confluence, cells were incubated overnight in serum-free medium then treated with myostatin. DNA was immunoprecipitated with c-Jun antibody and purified, extracted with phenol–chloroform, and subjected to RT-PCR. Agarose gel electrophoresis (1.5%) visualized PCR products under ultraviolet light. The IL-1β promoter region was amplified by the RT-PCR primers 5′-TCTGGTTCATCCATGAGATT-3′ and 5′-GGATAAAATGGGTACAATGAA-3′.

### Ethics Statement

Animal procedures were conducted according to approved protocols issued by the Institutional Animal Care and Use Committee at China Medical University (Taichung, Taiwan) (IACUC Approval No. 104-154-N). The study protocol for clinical sample collection was approved by the Institutional Review Board of China Medical University Hospital (CMUH104-REC2-055). All patients completed written informed consent prior to study entry.

### CIA Mouse Model

C57BL/6J mice, age: 8–10 weeks, obtained from the National Laboratory Animal Centre in Taipei, were maintained according to animal welfare conditions approved by China Medical University. Six mice formed the control group; the remaining mice were allocated to the CIA group. CIA was induced according to published protocols (23). Within 6 weeks after the primary immunization, 95% of the CIA group had developed severe arthritis. At 6 weeks, clinical severity was assessed in each knee by plethysmometer measurements.

### Immunohistochemistry

8-µm sections were prepared from paraffin-embedded tissues, deparaffinized in xylene, rehydrated in a graded alcohol series, and washed in de-ionized water. The antigens were retrieved in boiling 10 mM sodium citrate, pH 6.0 for 12 min; intrinsic peroxidase activity was blocked by incubation with 3% hydrogen peroxide. 3% BSA in PBS blocked nonspecific antibody-binding sites. Sections were incubated overnight at 4°C in diluted primary antibodies specific for myostatin or IL-1β. After PBST washing, the secondary antibody (HRP-labeled anti-rabbit IgG) was applied for 30 min at room temperature. Stained sections were examined with 3,3′-diaminobenzidine tetrahydrochloride, counterstained with hematoxylin and eosin and observed under a light microscope. Safranin O-fast Green staining of the same specimens determined bone erosion.

### Statistics

SigmaPlot software was used to quantify results, which are expressed as the mean ± SEM of at least three experiments. We used the Student’s *t*-test to compare the means between groups. For comparisons of more than two groups, we used two-factor analysis of variance with the Bonferroni *post hoc* test. Results were considered statistically significant when *p* < 0.05.

## Results

### High Expression of Myostatin and IL-1β in Synovial Fluid Samples from RA Patients

**Figure 1:**
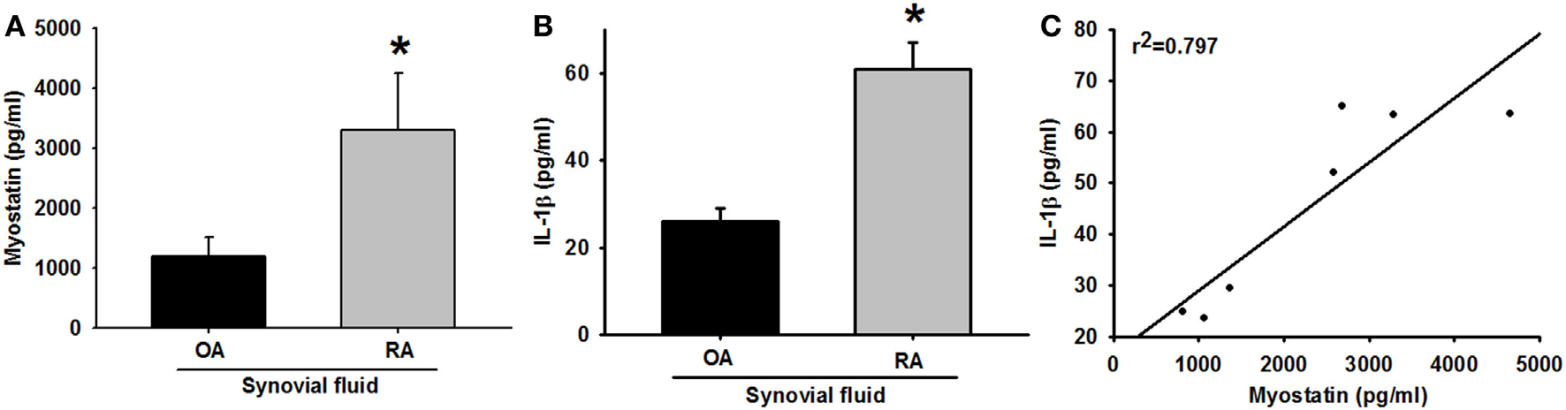
High expression of myostatin and interleukin (IL)-1β in rheumatoid arthritis (RA) synovial fluid. **(A,B)** The enzyme-linked immunosorbent assay was used to measure myostatin and IL-1β levels in synovial fluid from RA and osteoarthritis (OA) patients (*n* = 4). **(C)** A positive correlation between myostatin and IL-1β expression in synovial fluid from RA and OA patients. Results are expressed as the mean ± SEM. *p < 0.05 compared with OA patients (Student’s *t*-test).

Activated RASFs produce pro-inflammatory cytokines that infiltrate into synovial fluid and thereby initiate and perpetuate RA (24). Myostatin positively regulates IL-6, TNF-α, and IL-17 (18). We, therefore, analyzed myostatin and IL-1β expression profiles in human synovial fluids, using samples obtained during total knee arthroplasty from RA and OA patients. Levels of myostatin and IL-1β were significantly higher in RA synovial samples compared with OA samples (Figures 1A,B). Moreover, the expression patterns of myostatin and IL-1β were highly and positively correlated (Figure 1C). These observations suggest that myostatin and IL-1β might play a role in RA pathogenesis.

### Myostatin Promotes IL-1β Expression

**Figure 2:**
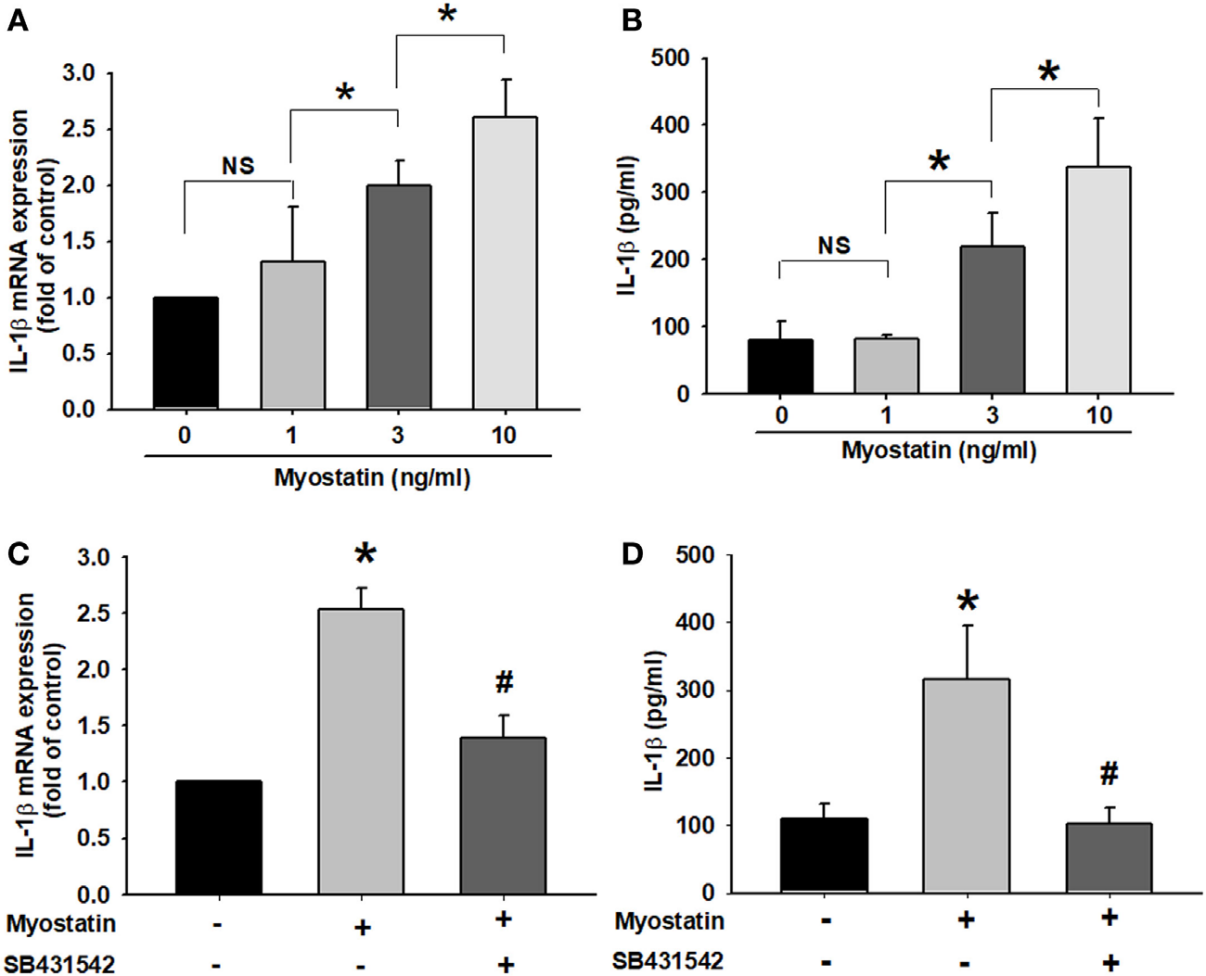
Myostatin induces interleukin (IL)-1β expression *via* the ALK receptor in RA synovial fibroblasts. **(A,B)** MH7A cells were treated with myostatin (0–10 ng/mL) for 24 h, then IL-1β mRNA expression (*n* = 3–4) (0, 1, 3 ng/mL: *n* = 4; 10 ng/mL: *n* = 3) and IL-1β protein secretion (*n* = 4–6) (0, 1, 10 ng/mL: *n* = 4; 3 ng/mL: *n* = 6) were evaluated by quantitative polymerase chain reaction (qPCR) and enzyme-linked immunosorbent assay (ELISA), respectively. **(C,D)** Cells were incubated with SB431542 (10 µM) for 30 min, then stimulated with myostatin (10 ng/mL) for 24 h. Cells were harvested and examined for IL-1β expression by qPCR (*n* = 4) and ELISA assays (*n* = 5). Results are expressed as the mean ± SEM. *p < 0.05 compared with controls. ^#^*p* < 0.05 compared with the myostatin-treated group [**(A,B)** Bonferroni *post hoc* test, **(C,D)** Student’s *t*-test].

To further characterize the role of myostatin inducing RASF activation *via* IL-1β expression, we examined IL-1β production after myostatin treatment in human MH7A cells. qPCR and ELISA assays revealed dose-dependent increases with myostatin in IL-1β mRNA expression and protein secretion (Figures 2A,B). However, treatment with myostatin for 6 or 12 h only slightly increased IL-1β mRNA expression (Figure S1 in Supplementary Material). The evidence demonstrates that myostatin binds to the activin type IIB receptor and then interacts with either ALK4 or ALK5 to stimulate downstream signaling (25, 26). When MH7A cells were pretreated with SB431542 (a potent ALK receptor inhibitor), myostatin-induced IL-1β expression was significantly reduced (Figures 2C,D). Thus, IL-1β expression is increased by myostatin *via* the ALK receptor.

### The Role of JNK and ERK Signaling Pathways in Myostatin-Stimulated IL-1β Expression

**Figure 3:**
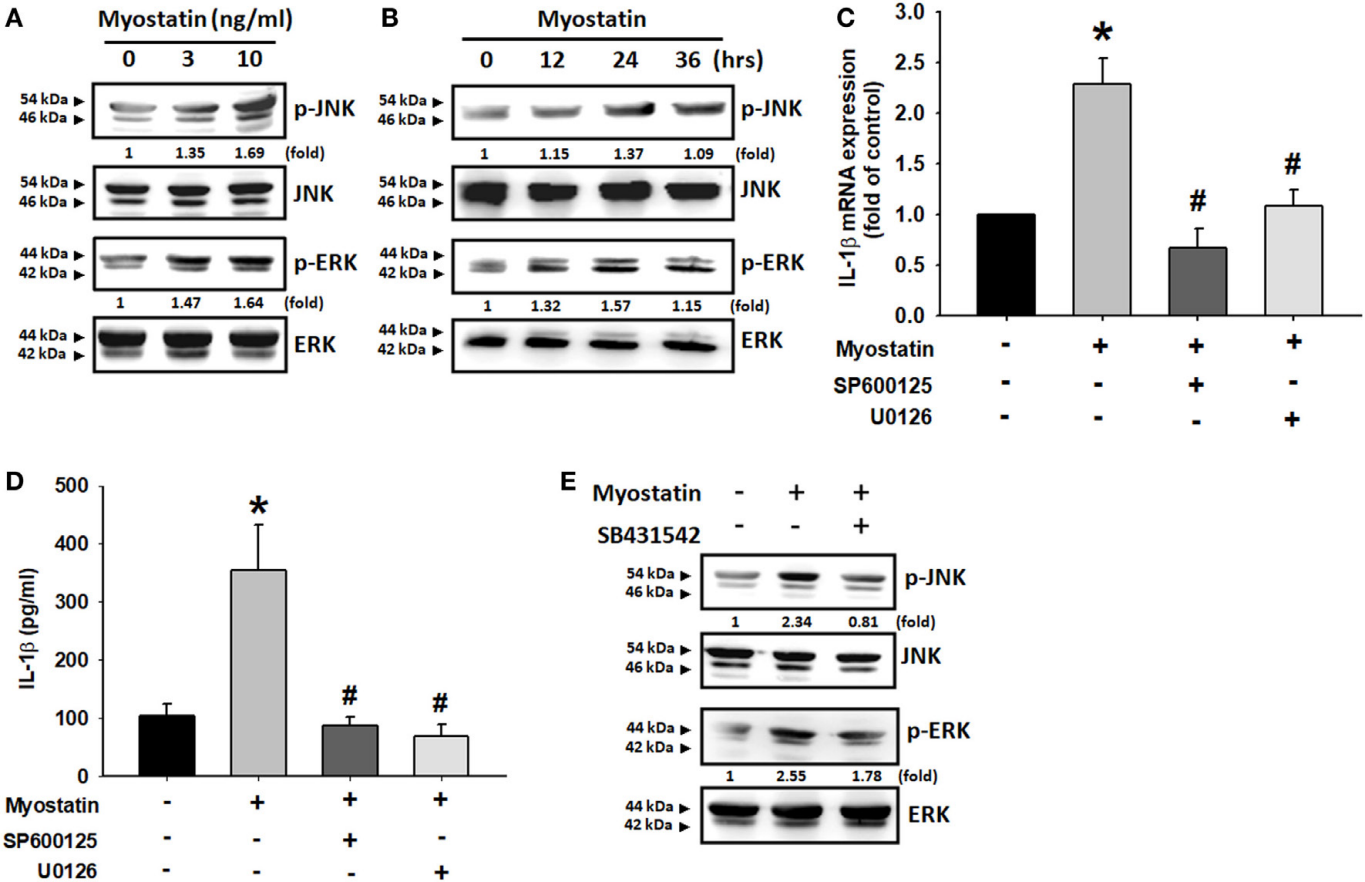
The JNK and ERK signaling pathways were involved in myostatin-induced interleukin (IL)-1β expression. **(A,B)** MH7A cells were incubated with various concentrations of myostatin (0–10 ng/mL) for 24 h or 10 ng/mL in indicated time intervals (*n* = 3); JNK and ERK phosphorylation was examined by western blot (*n* = 3). **(C,D)** MH7A cells were pretreated with SP600125 (10 µM) or U0126 (10 µM) for 24 h, prior to stimulation with myostatin for 24 h. IL-1β expression was examined by quantitative polymerase chain reaction (*n* = 3) and enzyme-linked immunosorbent assays (*n* = 3–4) (Myostatin: *n* = 3; Control, SP600125, U0126: *n* = 4). **(E)** MH7A cells were pretreated with SB431542 (10 µM) for 30 min then stimulated with myostatin (10 ng/mL) for 24 h, and JNK and ERK phosphorylation was examined by western blot (*n* = 3). Results are expressed as the mean ± SEM. *p < 0.05 compared with controls. ^#^*p* < 0.05 compared with the myostatin-treated group (Student’s *t*-test).

The ERK, p38, and JNK signaling pathways have been implicated as participating in myostatin signal transduction (26–28). We sought to determine the relative contributions of p38, JNK, and ERK activation upon myostatin-mediated regulation of IL-1β expression. Within the first 2 h of myostatin treatment, we found no evidence of p38, JNK, and ERK activation (data not shown). In contrast, an analysis of the expression of JNK and ERK proteins during prolonged myostatin treatment revealed that myostatin-promoted JNK and ERK phosphorylation in a dose- and time-dependent manner (Figures 3A,B), whereas no such effect was seen with p38 signaling (Figure S2 in Supplementary Material). We next observed the role of JNK and ERK in the mediation of myostatin-induced IL-1β expression, by using SP600125 (a broad-spectrum JNK inhibitor) and U0126 (a highly selective ERK inhibitor). Both agents abolished myostatin-induced increases in IL-1β expression (Figures 3C,D). Moreover, we confirmed a relationship between the ALK receptor and JNK/ERK signaling pathways and the myostatin pathway. Incubating the cells with the ALK receptor inhibitor reduced myostatin-induced increases in JNK and ERK phosphorylation (Figure 3E). These data demonstrate that myostatin induces IL-1β production in RASFs *via* the ALK receptor and JNK/ERK signaling pathways.

### AP-1 Is Involved in Myostatin-Induced IL-1β Expression

**Figure 4:**
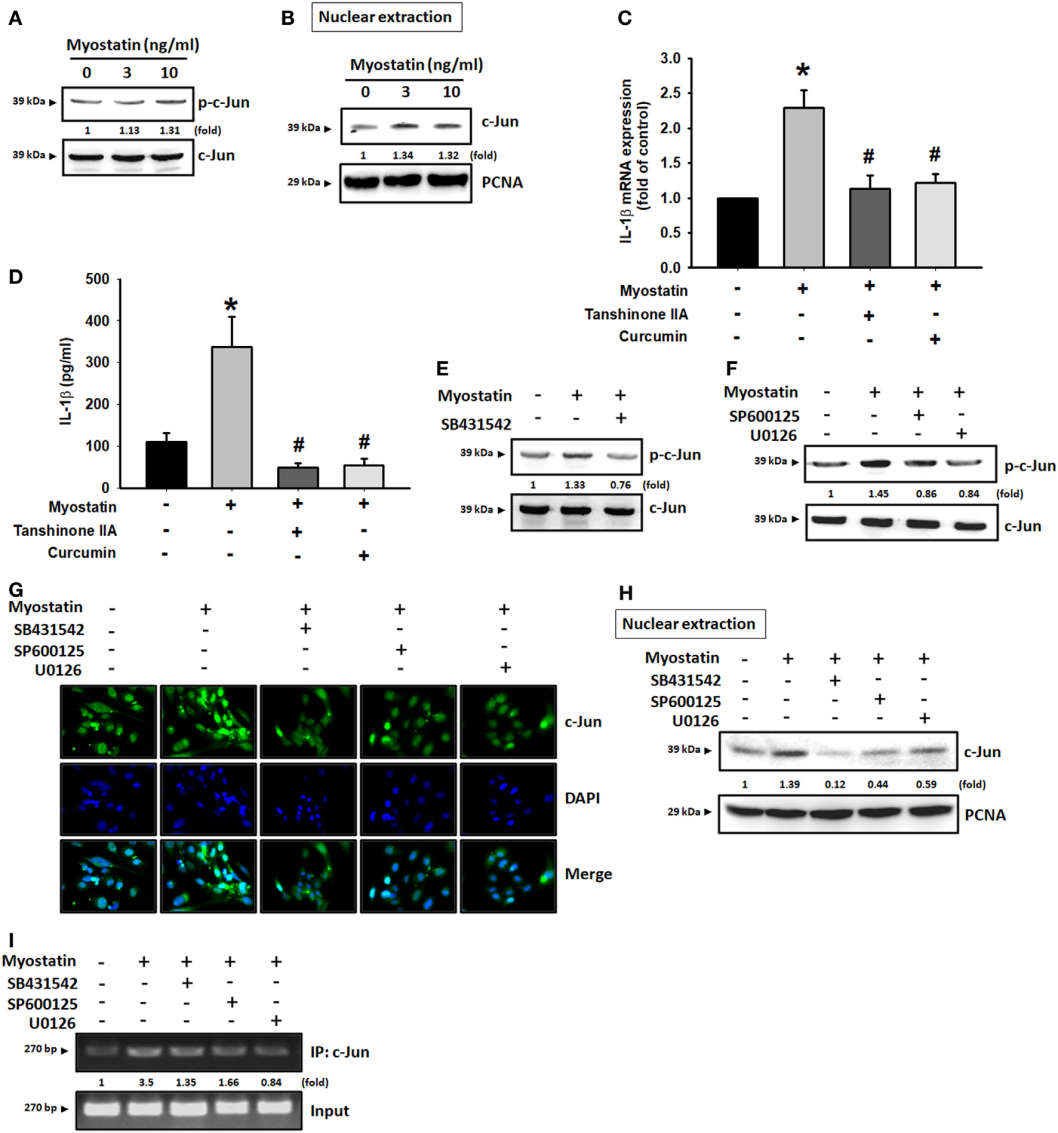
Involvement of AP-1 in myostatin-induced interleukin (IL)-1β production. **(A)** MH7A cells were incubated with various concentrations of myostatin (0–10 ng/mL) for 24 h; c-Jun phosphorylation was examined by western blot (*n* = 3). **(B)** MH7A cells were treated with various concentrations of myostatin (0–10 ng/mL) for 24 h and c-Jun expression in nuclear extracts was evaluated by western blot (*n* = 3). **(C,D)** MH7A cells were pretreated with Curcumin (10 µM) or Tanshinone IIA (10 µM) for 30 min, then stimulated with myostatin for 24 h, and IL-1β expression was examined by quantitative polymerase chain reaction (*n* = 3) and enzyme-linked immunosorbent assays (*n* = 4). **(E,F)** Cells were pretreated for 30 min with SB431542 (10 µM), SP600125 (10 µM), or U0126 (10 µM), prior to stimulation with myostatin for 24 h. c-Jun phosphorylation (*n* = 3) was examined by western blot (*n* = 3). **(G–I)** Cells were pretreated for 30 min with SB431542 (10 µM), SP600125 (10 µM), or U0126 (10 µM), prior to stimulation with myostatin for 24 h. c-Jun translocation into the nucleus was examined by immunofluorescence staining (*n* = 3) and western blot (*n* = 3); c-Jun binding to the AP-1 motif was examined by chromatin immunoprecipitation (*n* = 3). Results are expressed as the mean ± SEM. *p < 0.05 compared with controls. ^#^*p* < 0.05 compared with the myostatin-treated group (Student’s *t*-test).

The transcription factor AP-1 is a well-known downstream target of JNK and ERK (29–31). We, therefore, sought to determine whether myostatin disrupts AP-1 signaling. We found that incubating the cells with myostatin dose-dependently promoted c-Jun phosphorylation (Figure 4A). Moreover, when we separated nuclear extract after myostatin treatment, we observed that myostatin induced c-Jun translocation into the nucleus (Figure 4B). We then found that the AP-1 inhibitors curcumin and tanshinone IIA reduced myostatin-promoted IL-1β expression (Figures 4C,D). We also confirmed that the ALK receptor inhibitor, JNK inhibitor, and ERK inhibitor all reduced myostatin-promoted phosphorylation of c-Jun and c-Jun accumulation in the nuclei (Figures 4E–H). Results of a ChIP assay confirmed that c-Jun binds to the AP-1 binding site in the IL-1β promoter after myostatin stimulation, and this was attenuated by inhibition of the ALK receptor and JNK/ERK signaling (Figure 4I). These data suggest that myostatin not only acts through the ALK receptor and JNK, ERK, AP-1 signaling pathways but also promotes IL-1β expression in RASFs.

### Increased IL-1β Production *via* Downregulation of Myostatin-Mediated miR-21 Expression

**Figure 5:**
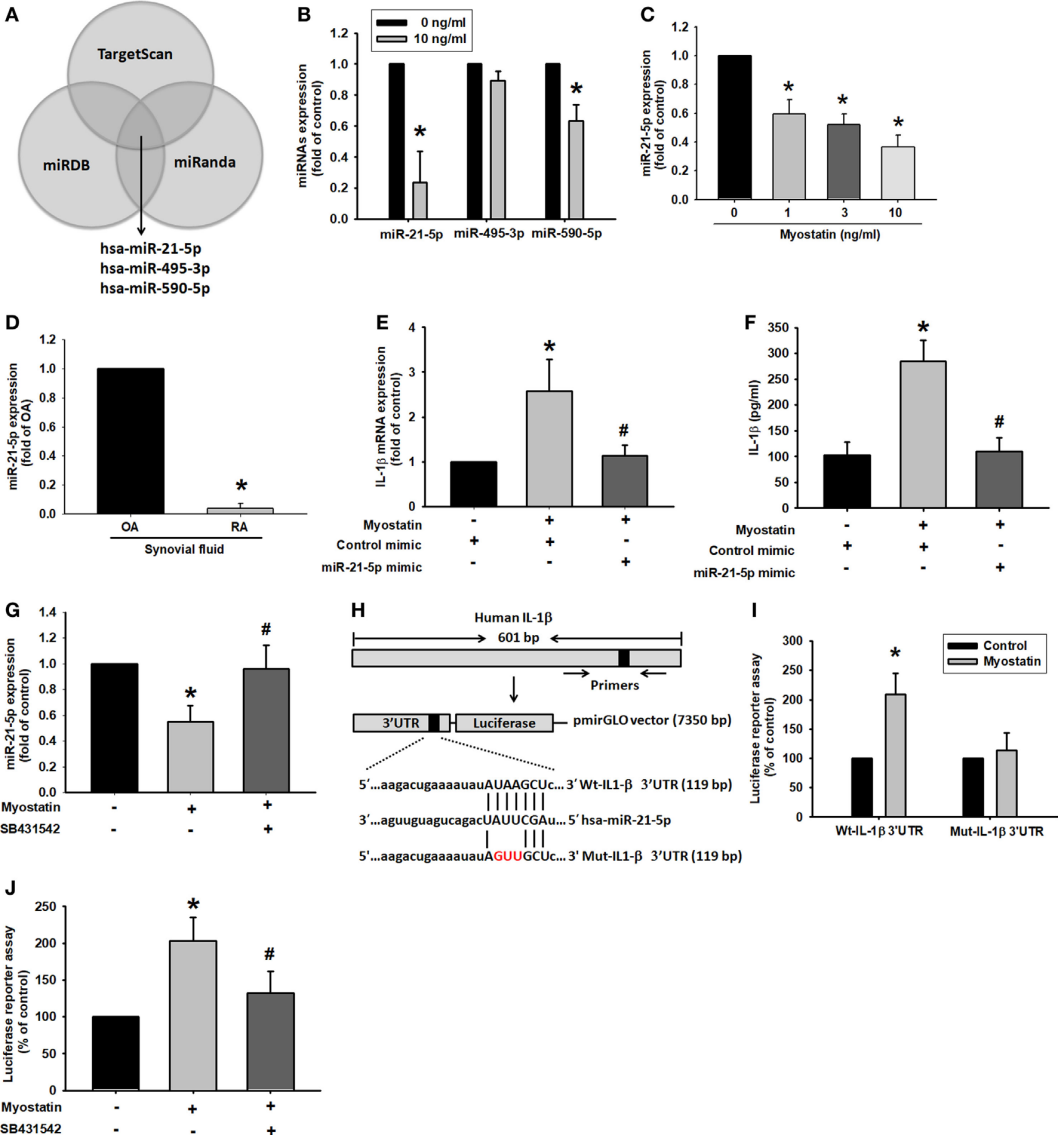
miR-21-5p inhibition is involved in myostatin-induced interleukin (IL)-1β expression. **(A)** Use of TargetScan, miRDB, and miRanda computational software to identify miRNAs that potentially bind to the IL-1β 3′ UTR. **(B,C)** Cells were stimulated with myostatin for 24 h, and miRNA expression was examined by quantitative polymerase chain reaction (qPCR) (*n* = 3). **(D)** miR-21-5p expression in rheumatoid arthritis (RA) or osteoarthritis (OA) synovial fluid was measured by qPCR (*n* = 4). **(E,F)** Cells were transfected with miR-21-5p mimic for 24 h then stimulated with myostatin for 24 h. IL-1β expression was examined by qPCR (*n* = 3) and enzyme-linked immunosorbent assays (*n* = 4–5) (Myostatin: *n* = 4; control mimic, miR-21-5p: *n* = 5). **(G)** Cells were pretreated for 30 min with SB431542 then stimulated with myostatin for 24 h. miR-21-5p expression was examined by qPCR (*n* = 3–4) (Myostatin: *n* = 3; Control, SB431542: *n* = 4). **(H)** Schematic 3′ UTR representation of human IL-1β containing the miR-21-5p binding site. **(I,J)** Cells were transfected with a wt-IL-1β-3′ UTR or mut-IL-1β-3′ UTR plasmid for 24 h then stimulated with myostatin for 24 h; relative luciferase/renilla activities were then measured (*n* = 3). Results are expressed as the mean ± SEM. *p < 0.05 compared with controls. ^#^*p* < 0.05 compared with the myostatin-treated group (Student’s *t*-test).

miRNAs are involved in classical epigenetic mechanisms, such as downregulation of pro-inflammatory expression at the posttranscriptional level (10, 11, 32, 33). Most importantly, miRNAs have been identified as having therapeutic potential in arthritic diseases (34). To understand the mechanism underlying miRNA-mediated myostatin promotion of IL-1β expression, we used three target prediction programs (TargetScan, miRDB, and miRanda) and identified potential miRNA binding sites involving the IL-1β 3′ UTR sequence (Figure 5A). We found that myostatin dose-dependently downregulated miR-21-5p (Figures 5B,C). Notably, miR-21-5p levels were much lower in RA than in OA synovial fluid (Figure 5D). Next, we used the miR-21-5p mimic to investigate miR-21-5p involvement in myostatin-enhanced IL-1β expression. Transfection of cells with the miR-21-5p mimic weakened myostatin-induced IL-1β mRNA expression and protein secretion (Figures 5E,F). Pretreatment of cells with SB431542 significantly reversed myostatin-induced inhibition of miR-21-5p expression (Figure 5G). To examine whether miR-21-5p mediates the IL-1β 3′ UTR sequence, we constructed a luciferase reporter vector harboring this sequence that contained the miR-21-5p binding site (Figure 5H). Luciferase activity was increased by myostatin in the wild-type (wt)-IL-1β 3′ UTR plasmid; no such change was seen with the mutant (mut)-IL-1β 3′ UTR plasmid, while the ALK receptor inhibitor reduced myostatin-mediated wt-IL-1β 3′ UTR activity (Figures 5I,J). These data suggest that miR-21-5p directly targets IL-1β and thus downregulates IL-1β expression.

### Correlation of Myostatin and IL-1β in the CIA Mouse Model

**Figure 6:**
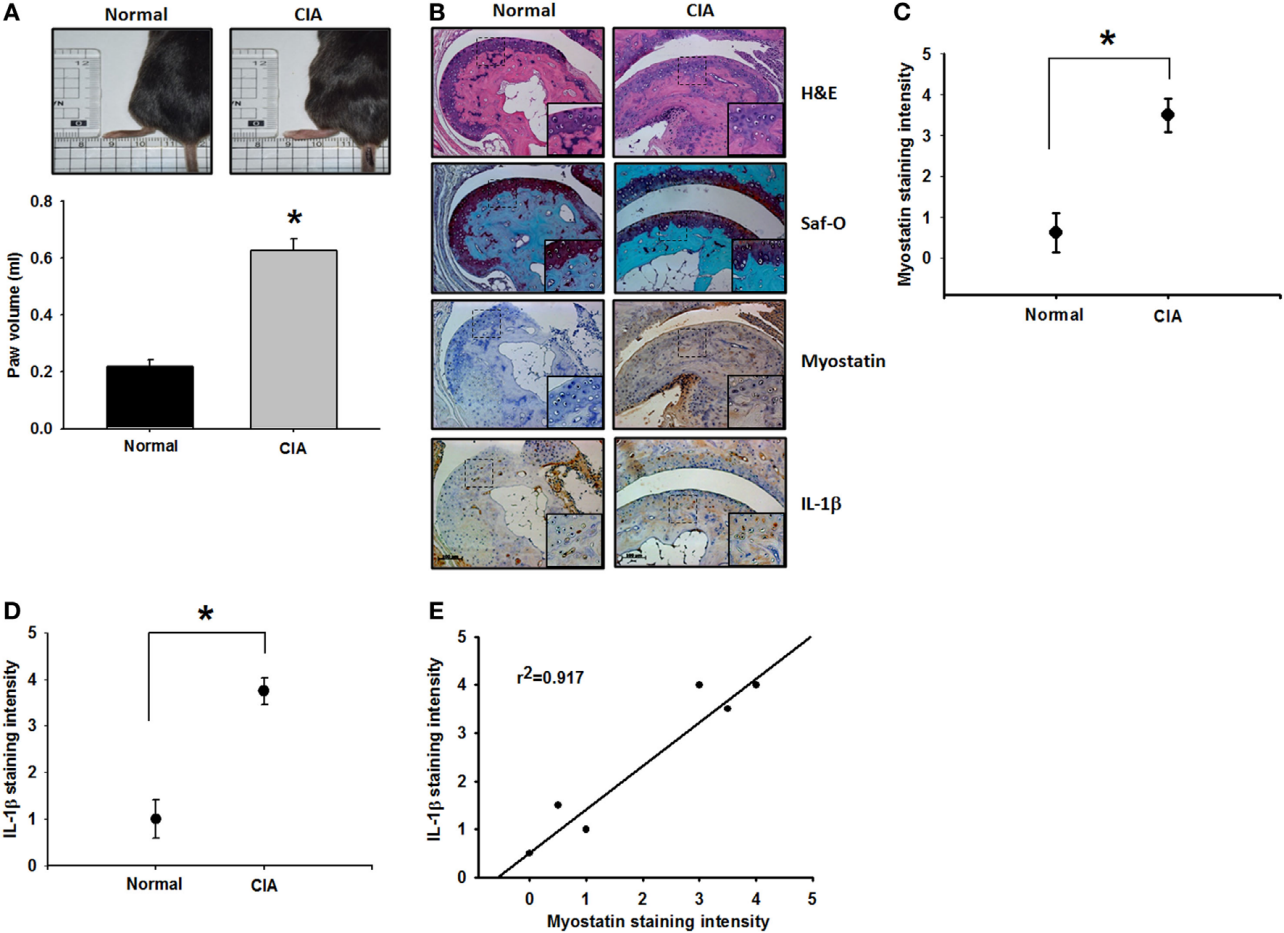
Myostatin and interleukin (IL)-1β overexpression in the collagen-induced arthritis (CIA) mouse model. **(A)** Hind paw swelling was photographed and measured with a digital plethysmometer in healthy controls and CIA mice (*n* = 4–5) (Control: *n* = 4; CIA: *n* = 5). **(B)** Histologic sections of ankle joints were stained with H&E or Safranin O and immunostained with myostatin or IL-1β (*n* = 4). **(C,D)** Quantification results. **(E)** A positive correlation between myostatin and IL-1β in CIA mice (*n* = 4). Results are expressed as the mean ± SEM. *p < 0.05 compared with controls. ^#^*p* < 0.05 compared with the myostatin-treated group (Student’s *t*-test).

Paw swelling was significantly pronounced in CIA mice compared with controls (Figure 6A). IHC staining of murine tissue revealed substantially higher levels of myostatin and IL-1β expression from CIA mice compared with controls (Figures 6B–D). IHC data also revealed a high, positive correlation between myostatin and IL-1β (Figure 6E). These findings indicate that myostatin and IL-1β expression influence the progression of RA disease.

## Discussion

Rheumatoid arthritis is characterized by RASFs and complex cellular interactions among cytokines and growth factors that lead to progressive destruction of joints, ultimately resulting in disability and loss of function (1, 35–38). IL-1β has been identified as being a major contributor to RASF inflammation (39). In this study, we found that high levels of IL-1β and myostatin in human RA synovial fluid influence RASF inflammation. Specifically, we found that myostatin induces IL-1β expression through the ALK receptor and JNK, ERK, and AP-1 signaling pathways, as well as *via* downregulation of miR-21-5p expression.

Interleukin-1β is a pivotal pro-inflammatory cytokine that mediates the systemic inflammatory response and is implicated in the pathogenesis of many diseases, such as RA (40–42). It is well known that IL-1β and the IL-1R are abundant in RA synovium (43); the anti-IL-1β neutralizing monoclonal antibody canakinumab and the recombinant human IL-1R antagonist anakinra show promise in the treatment of RA (7, 8, 44, 45). However, the mechanism underlying the secretion of IL-1β from RASFs and resultant inflammation remains largely unclear. Thus, we searched for an upstream mediator of IL-1β in RASFs, which are often associated with rheumatoid cachexia (43). Myostatin is highly expressed in synovial membranes and inhibition of myostatin expression improves skeletal muscle atrophy in RA disease (20, 46). Myostatin negatively modulates myogenesis and mutations in the myostatin gene reduce levels of mature protein in mice (16, 47). Most importantly, myostatin acts as a regulator of pro-inflammatory factors in some diseases, e.g., chronic kidney disease and type 2 diabetes mellitus (18, 19). In our study, we found that application of myostatin to RASFs dose-dependently induced IL-1β expression. Notably, myostatin and IL-1β levels in human RA synovial fluid and from CIA murine tissue were over-expressed and positively correlated. These findings indicate that myostatin directly promotes inflammation in RASFs *via* IL-1β upregulation. This report is the first study to specifically identify the function of myostatin in RASFs.

Several reports have documented that myostatin functions through multiple signaling pathways (48, 49). However, its association with MAPKs in the regulation of RASF inflammation is uncertain. MAPKs, consisting of the ERK, JNK, and p38 MAPK cascades, transduce extracellular signals into cells and play an important role in disease (50, 51). Increases in ERK and JNK phosphorylation are seen in early arthritis and in erosive disease (52). Here, we found that myostatin significantly induces JNK and ERK activation in RASFs. Pretreatment with JNK and ERK inhibitors attenuated myostatin-induced IL-1β expression. Strikingly, p38 activity has no effect upon phosphorylation after myostatin stimulation (Figure S2 in Supplementary Material). Some evidence demonstrates that p38 activity is neither affected in TNF-transgenic mice nor in patients with psoriatic arthritis (PsA) (53, 54). According to these findings, p38 may not be involved in myostatin-mediated inflammatory responses in RA.

Interestingly, we also identified differentially expressed miRNAs whose function has not previously been implicated in myostatin-induced regulation of RASF inflammation. Using computational software to predict miRNAs, we identified miR-21-5p sequences complementary to the IL-1β 3′ UTR sequence that were downregulated by myostatin stimulation. Moreover, treatment of cells with miR-21-5p mimic reduced myostatin-induced IL-1β expression. These data indicate that myostatin induces IL-1β expression not only through the JNK and ERK pathways and AP-1 transcription factors but also *via* downregulation of miR-21-5p expression. The prediction software showed that miR-21-5p may also bind to the JNK 3′ UTR, but there are no published reports on this aspect. Further investigation is needed into the role of myostatin in the activation of RASF. Our finding of a low level of miR-21-5p expression has been both supported and contradicted by the results of two recently published papers that investigated the role of miR-21-5p in inflammation/arthritis models (15, 55). The first paper reports significantly downregulated expression of miR-21-5p in patients with RA and in centenarians, compared with healthy controls. The study authors suggest that the low miR-21-5p expression in their RA cohort might be due to corticosteroid therapy, which inhibits the nuclear factor-κB and its absence blocks the expression of miR-21-5p (15). In contrast, the second paper describes a significant upregulation of miR-21-5p expression in patients with early-onset PsA or early RA, as compared with healthy controls. The study authors conclude that this upregulation of miR-21-5p highlights its role in the inflammatory process in general and that it is reasonable to target this miRNA in different inflammatory disorders (55). The findings of these two papers, in relation to our results, underline the fact that we need to confirm more about the apparent links between miRNA expression and corticosteroid therapy and autoimmune disease.

**Figure 7:**
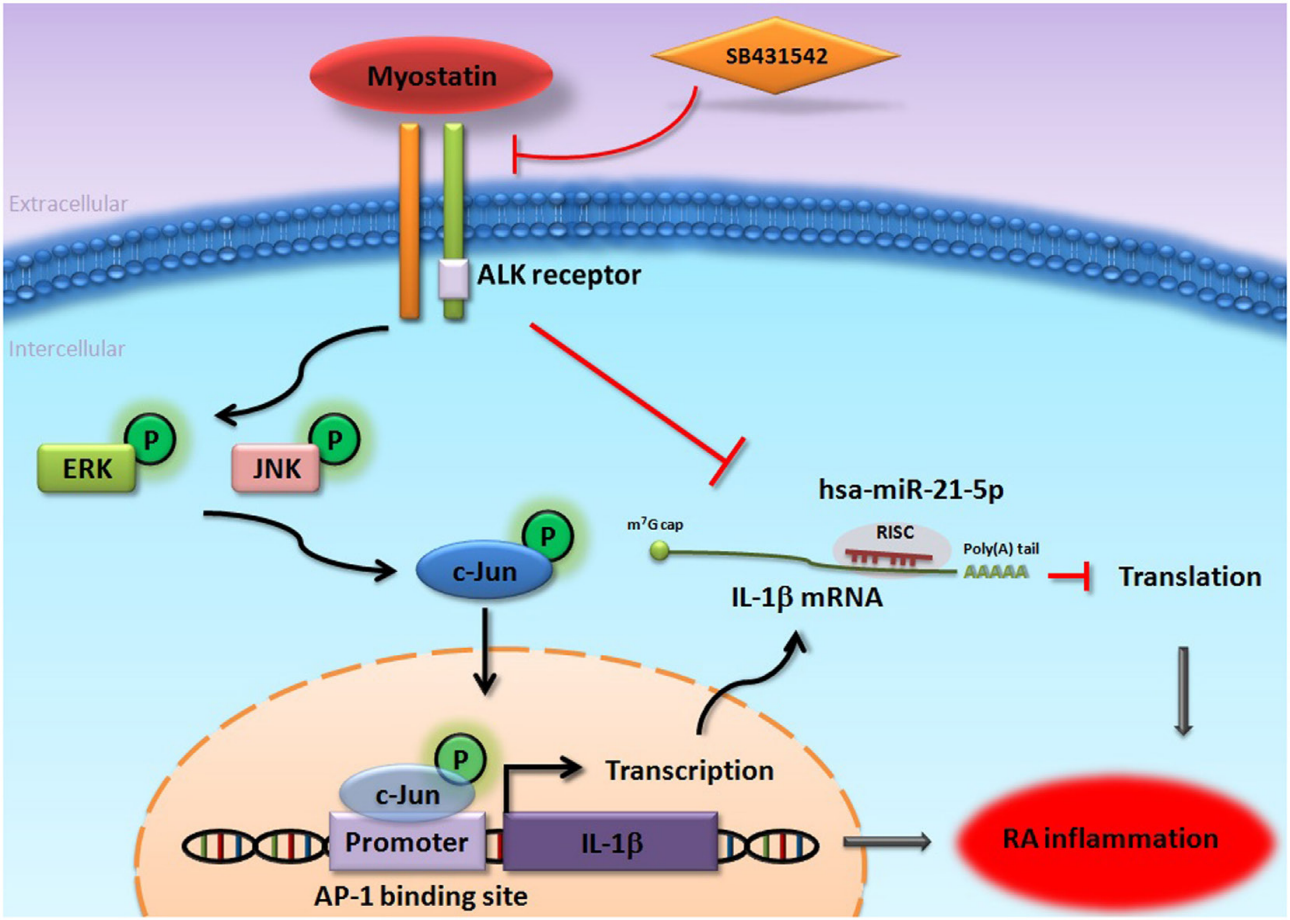
Schema of myostatin-induced promotion of interleukin (IL)-1β expression in RA synovial fibroblasts (RASFs). Myostatin induces IL-1β expression in RASFs through the ALK receptor, JNK, ERK, and AP-1 signaling pathways, as well as *via* downregulation of miR-21-5p expression.

In summary, we demonstrate that myostatin induces IL-1β expression through the ALK receptor, JNK, ERK, and AP-1 signaling pathways, as well as *via* downregulation of miR-21-5p expression (Figure 7). According to our findings, the inhibition of myostatin or overexpression of miR-21-5p may be relevant therapeutic targets in RA.

## Ethics Statement

Animal procedures were conducted according to approved protocols issued by the Institutional Animal Care and Use Committee at China Medical University (Taichung, Taiwan) (IACUC Approval No. 104-154-N). The study protocol for clinical sample collection was approved by the Institutional Review Board of China Medical University Hospital (CMUH104-REC2-055). All patients completed written informed consent prior to study entry.

## Author Contributions

Study design: S-LH, A-CC, C-CL, C-HTang. Acquisition, analysis, and interpretation of data and statistical analysis: S-LH, A-CC, C-CH, C-HTsai. Manuscript preparation: S-LH, A-CC, C-HTang.

## Conflict of Interest Statement

The authors declare that the research was conducted in the absence of any commercial or financial relationships that could be construed as a potential conflict of interest.
