# Supplementary material for: Myostatin Promotes Interleukin-1β Expression in Rheumatoid Arthritis Synovial Fibroblasts through Inhibition of miR-21-5p
Source: Front Immunol. 2017 Dec 8;8:1747. doi: 10.3389/fimmu.2017.01747 (PMC5727021; doi:10.3389/fimmu.2017.01747)
Supplement: Supplementary file 1 [file Image_1.PDF]

Supplementary data

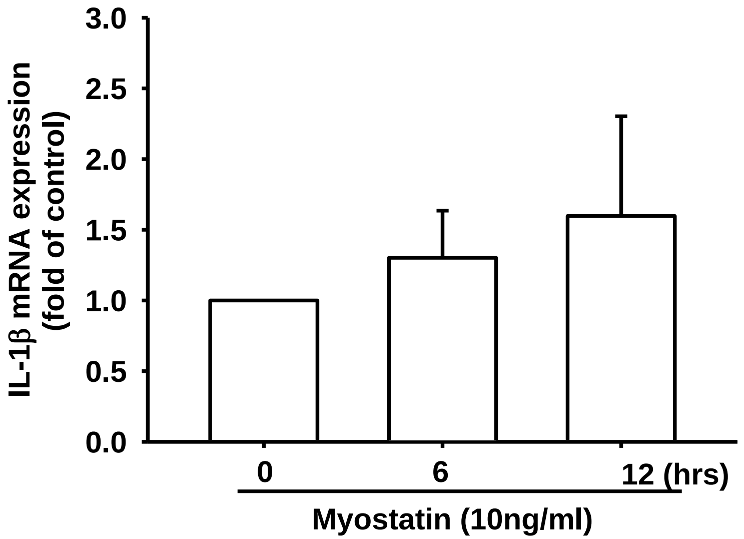

Figure S1: Analysis of IL-1β mRNA expression at particular time points.

MH7A cells were treated with myostatin (10 ng/mL) at the indicated time intervals, then IL-1β mRNA expression was evaluated by qPCR assay (n = 4).

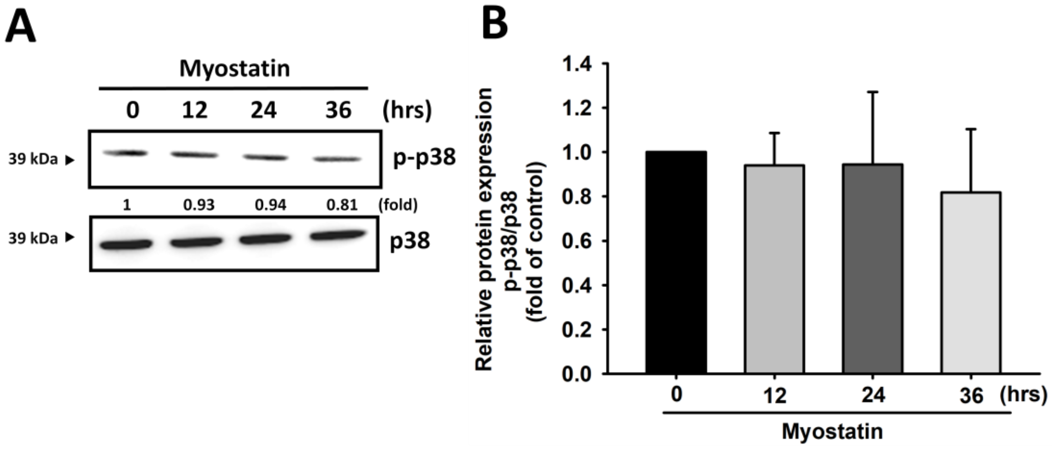

Figure S2: p38 activity has no effect upon phosphorylation during myostatin treatment. (A) MH7A cells were incubated with myostatin (10 ng/mL) at the indicated time intervals; p38 phosphorylation was examined by Western blot. (B) Quantification of p38 phosphorylation normalized with p38 (n = 3).

Fig.3A

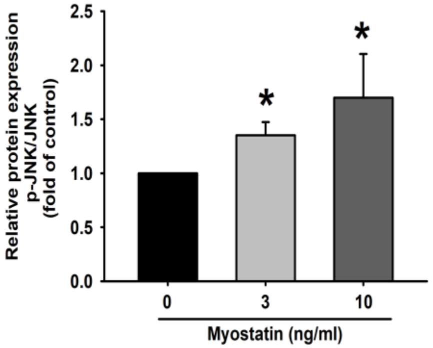

Fig.3A

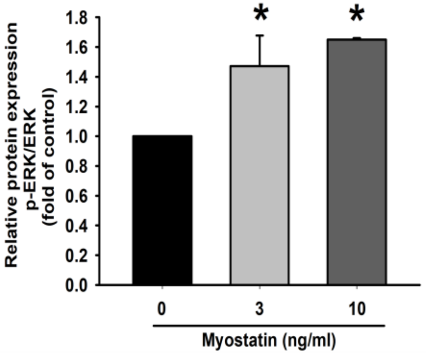

Fig.3B

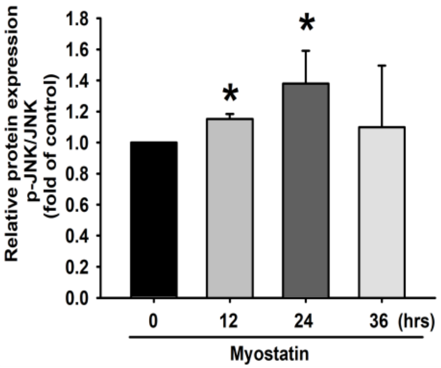

Fig.3B

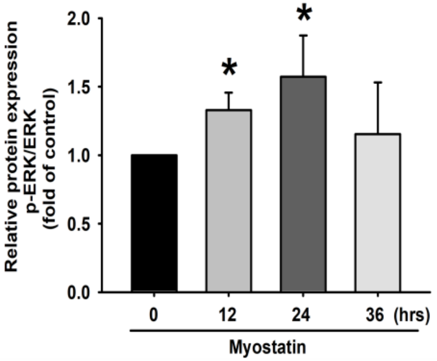

Fig.3E

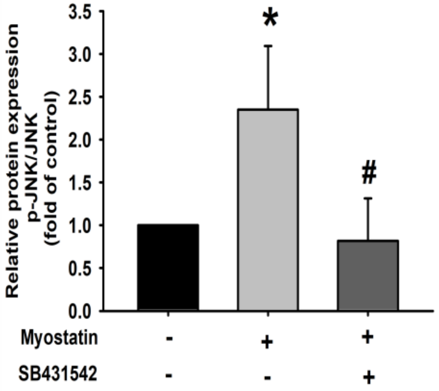

Fig.3E

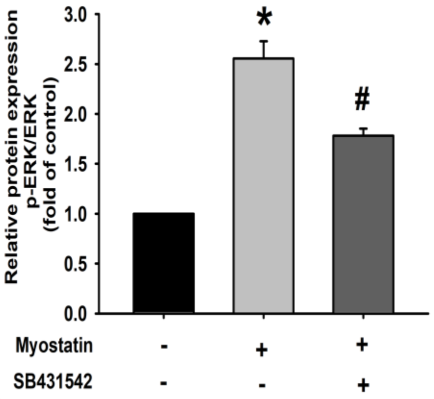

Fig.4A

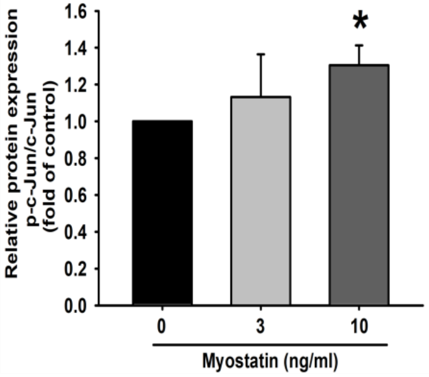

Fig.4B

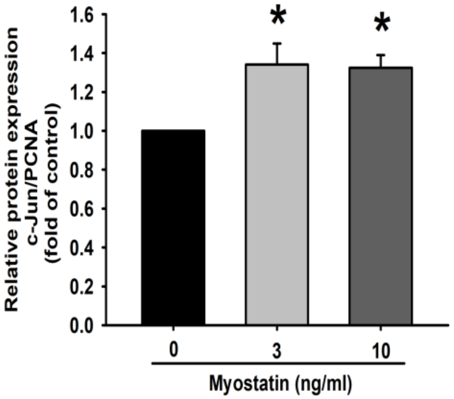

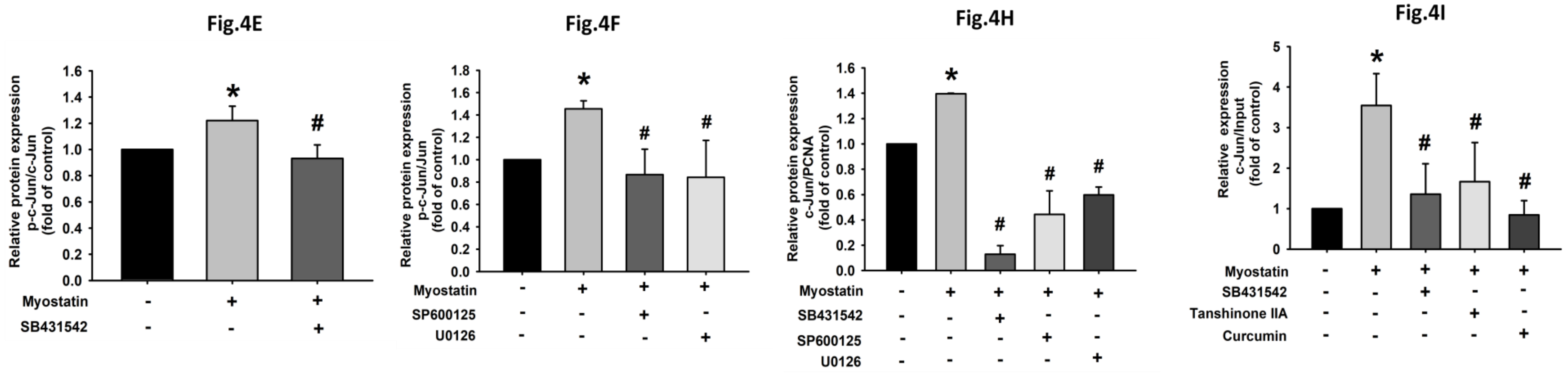

Figure S3: Densitometry analysis of protein and DNA expression. Results are expressed as the mean ± S.E.M. *$p < 0.05$ compared with controls. #$p < 0.05$ compared with the myostatin-treated group (Student's t-test).
